# Visceral fat area is a strong predictor of leukocyte cell-derived chemotaxin 2, a potential biomarker of dyslipidemia

**DOI:** 10.1371/journal.pone.0173310

**Published:** 2017-03-09

**Authors:** Kumpei Tanisawa, Hirokazu Taniguchi, Xiaomin Sun, Tomoko Ito, Ryoko Kawakami, Shizuo Sakamoto, Mitsuru Higuchi

**Affiliations:** 1 Department of Health Promotion and Exercise, National Institutes of Biomedical Innovation, Health and Nutrition, Tokyo, Japan; 2 Research Fellow of Japan Society for the Promotion of Science, Tokyo, Japan; 3 Faculty of Sport Sciences, Waseda University, Tokorozawa, Japan; 4 Institute of Advanced Active Aging Research, Waseda University, Tokorozawa, Japan; 5 Faculty of Agriculture, Ryukoku University, Shiga, Japan; 6 School of Public Health, Xi'an Jiaotong University, Xi'an, Shaanxi, China; 7 Graduate School of Sport Sciences, Waseda University. Tokorozawa, Japan; Rutgers New Jersey Medical School, UNITED STATES

## Abstract

**Background:**

Leukocyte cell-derived chemotaxin 2 (LECT2) is a hepatokine linking obesity to skeletal muscle insulin resistance. Although previous studies reported that obesity was associated with high levels of circulating LECT2 in human, the associations of detailed body fat distribution with LECT2 levels have not been examined. Furthermore, although animal study suggested that exercise decreased circulating LECT2 levels, it remains unknown whether physical fitness is associated with LECT2 levels in human. We therefore examined the relationship of plasma LECT2 levels with various adiposity indices and cardiorespiratory fitness (CRF) in middle-aged and elderly Japanese men. Furthermore, we examined the relationship of LECT2 levels with the presence of metabolic syndrome, hypertension, insulin resistance and dyslipidemia to determine the clinical significance of measuring circulating LECT2.

**Materials and methods:**

This was a cross-sectional study of 143 Japanese men (age: 30–79 years). Participants’ plasma LECT2 levels were measured by an enzyme-linked immunosorbent assay. To assess their abdominal fat distributions, visceral fat area (VFA) and subcutaneous fat area (SFA) were measured using magnetic resonance imaging. CRF was assessed by measuring peak oxygen uptake ($\dot{V}O_{2}\mathrm{peak}$).

**Results:**

All adiposity indices measured in this study were positively correlated with plasma LECT2 levels, while $\dot{V}O_{2}\mathrm{peak}$ was negatively correlated with LECT2 levels after adjustment for age. The correlations, except for VFA were no longer significant with further adjustment for VFA. Stepwise multiple linear regression analysis revealed that VFA was the strongest predictor of plasma LECT2 levels. Plasma LECT2 levels differed based on the presence of metabolic syndrome and dyslipidemia, but not hypertension and insulin resistance. Logistic regression analyses revealed that plasma LECT2 levels were significantly associated with dyslipidemia independently of VFA; VFA was not significantly associated with dyslipidemia after adjustment for LECT2.

**Conclusion:**

VFA was the strongest predictor of plasma LECT2 that is a potential biomarker linking visceral obesity to dyslipidemia.

## Introduction

Recent studies have demonstrated that the liver is a secretory organ that produces several secretory proteins, named hepatokines [1]. Hepatokines such as fibroblast growth factor 21 (FGF21) [2], fetuin-A [3], and selenoprotein P [4] are predominantly expressed in the liver, and have been shown to function as metabolic regulators controlling energy homeostasis and glucose and lipid metabolism [5–8].

Recently, Lan et al. have revealed that leukocyte cell-derived chemotaxin 2 (LECT2) is a hepatokine linking obesity to skeletal muscle insulin resistance [9]. They showed that mice fed a high-fat diet exhibited increased body weight as well as elevated expression of *Lect2* in the liver and increased serum LECT2 levels, which were possibly regulated by decreased phosphorylation and activity of adenosine monophosphate-activated protein kinase (AMPK) in the liver. Furthermore, *Lect2* deletion attenuated skeletal muscle insulin resistance in mice fed a high-fat diet via dephosphorylation of the Jun NH2-terminal kinase [9]. Cross-sectional studies in humans have also demonstrated that serum LECT2 levels were positively correlated with body mass index (BMI), waist circumference (WC), and homeostasis model assessment-insulin resistance (HOMA-IR) [9], suggesting that LECT2 possibly plays a role in the development of obesity-induced insulin resistance even in humans. However, the relationships between detailed measurements of body fat distribution such as abdominal visceral and subcutaneous fat distributions and circulating LECT2 levels have not been examined so far. Because previous studies have indicated that visceral fat accumulation is more strongly associated with an adverse metabolic risk profile than subcutaneous accumulation [10], visceral fat area (VFA) may be strong predictor of circulating LECT2 levels.

Although obesity is a likely key determinant of LECT2 production in the liver, Lan et al. have also reported that exercise negatively regulates LECT2 expression [9]. They showed that acute running exercise in mice increased phosphorylation and activity of AMPK in the liver, thereby decreasing *Lect2* expression in the liver as well as serum LECT2 levels. Because many lines of evidence indicate that regular exercise increases cardiorespiratory fitness (CRF) and reduces the risk of insulin resistance and type 2 diabetes independently of adiposity indices [11–14], LECT2 may be a candidate hepatokine that links regular exercise or high CRF to improved insulin resistance. However, it remains unknown whether CRF is associated with LECT2 level independently of adiposity indices in humans.

In the present study, we performed a cross-sectional study to examine the relationship of plasma LECT2 levels with various adiposity indices and CRF in middle-aged and elderly Japanese men. Furthermore, we examined the relationship of LECT2 levels with the presence of metabolic syndrome, hypertension, insulin resistance and dyslipidemia to demonstrate the clinical significance of measuring circulating LECT2.

## Materials and methods

### Participants

The participants of the current study were 143 Japanese men (age: 30–79 years). Participants were originally recruited from 2012 to 2013 for a separate cross-sectional study examining the effects of aging and exercise on the relationship between genetic factors and metabolic syndrome risk in men [15, 16]; samples obtained during that study were reanalyzed in the present study. All participants were recruited from the general population of local communities through advertisement. The original cross-sectional study consisted of 210 men aged 20–79 years, and we excluded those participants 1) with a history of diabetes, cardiovascular disease, cancer, or chronic kidney failure (n = 18); 2) who were younger than 30 years of age (n = 22); 3) who had extremely high levels of serum triglycerides (≥1000 mg/dL) or low levels of low-density lipoprotein (LDL) cholesterol (<20 mg/dL) (n = 2); 4) with missing data (n = 7); and 5) who lacked plasma samples that had never been subjected to freeze thaw cycles (n = 18). Their current and former smoking status was recorded using a questionnaire. Daily alcohol intake was assessed using a brief self-administered diet history questionnaire. Fifty individuals (35.0%) reported regularly taking medications such as anti-hypertensive (n = 33/143, 23.1%), lipid-lowering (n = 10/143, 7.0%), gout suppressing (n = 4/143, 2.8%), or prostatic hyperplasia (n = 3/143, 2.1%) drugs. Eighty-five participants (59.4%) had hypertension, defined as: systolic blood pressure (SBP) ≥140 mmHg; diastolic blood pressure (DBP) ≥90 mmHg; and/or the use of antihypertensive drugs. Fifty-eight participants (40.6%) had dyslipidemia, defined as: high-density lipoprotein (HDL) cholesterol level <40 mg/dL; LDL cholesterol level ≥140 mg/dL; and/or triglyceride level ≥150 mg/dL. Another 7 participants (4.9%) had insulin resistance, defined as HOMA-IR ≥2.5. Seventeen participants (11.9%) had metabolic syndrome, defined as WC ≥85.0 cm combined with two or more of the following risk factors: SBP ≥130 mmHg and/or DBP ≥85 mmHg; triglyceride level ≥150 mg/dL and/or HDL cholesterol level <40 mg/dL; and/or fasting glucose level ≥110 mg/dL, as per the diagnostic criteria in Japan [17, 18]. The characteristics of the participants in the study are shown in Table 1.

**Table 1:** Characteristics of Participants.

*n*	143		
Age (year)	64.0 (56.0–69.0)		
Height (cm)	170.2	±	6.3
Body weight (kg)	68.6	±	9.3
BMI (kg/m^2^)	23.6	±	2.5
Body fat (%)	20.5	±	4.5
WC (cm)	84.1	±	7.6
VFA (cm^2^)	107.2	±	45.5
SFA (cm^2^)	104.2 (80.6–138.6)		
$\dot{V}O_{2}\mathrm{peak}$ (mL/kg/min)	33.1	±	7.2
SBP (mmHg)	135.0 (127.0–153.0)		
DBP (mmHg)	89.0 (83.0–98.0)		
AST (U/mL)	24.0 (21.0–27.3)		
ALT (U/mL)	20.0 (15.8–26.0)		
γ-GTP (U/mL)	28.0 (22.0–42.0)		
HDL cholesterol (mg/dL)	59.0 (50.8–68.0)		
LDL cholesterol (mg/dL)	121.6	±	28.9
Triglycerides (mg/dL)	91.0 (66.0–119.0)		
Fasting glucose (mg/dL)	95.8	±	8.9
Fasting insulin (μU/mL)	4.8 (3.6–6.6)		
HOMA-IR	1.2 (0.8–1.7)		
TNF-α (ng/mL)	1.0 (0.8–1.3)		
MCP-1 (ng/mL)	317.7 (271.2–386.9)		
LECT2 (ng/mL)	16.2 (13.7–18.9)		
Alcohol intake (g/day)	17.9 (6.0–41.3)		
Current/former smoking status (%)	48.3		
Metabolic syndrome (%)	11.9		
Hypertension (%)	59.4		
Dyslipidemia (%)	40.6		
Insulin resistance (%)	4.9		
Anti-hypertensive drug use (%)	23.1		
Lipid-lowering drug use (%)	7.0		
Gout suppressing drug use (%)	2.8		
Prostatic hyperplasia drug use (%)	2.1		

Values are presented as mean ± standard deviation or median and interquartile range.

All the participants provided written informed consent before enrollment in the study. The study was approved by the ethical review committee at Waseda University and was conducted in accordance with the Declaration of Helsinki.

### Anthropometric characteristics

The body weight and body fat percentage (assessed by bioelectrical impedance analysis) of each participant were measured using an electronic scale (Inner Scan BC-600, Tanita Inc., Tokyo, Japan), and their height was measured with a stadiometer (YL-65, YAGAMI Inc., Nagoya, Japan). The BMI was calculated by using the body weight and height values. WC was measured at the umbilical region with an inelastic measuring tape.

The VFA and subcutaneous fat area (SFA) were measured by magnetic resonance imaging (Signa 1.5 T, General Electric Inc., Milwaukee, WI, US) as described previously [19]. The imaging conditions included a T1 weighted spin-echo and axial-plane sequence with a slice thickness of 10 mm, a repetition time of 140 ms, and an echo time of 12.3 ms. The cross-sectional area of the VFA and SFA at the umbilical level was determined using image-analysis software (Slice-o-matic 4.3 for Windows, Tomovision, Montreal, Canada). All analyses were performed by the same investigator to minimize bias. The coefficient of variation for the cross-sectional area at the umbilical level was 0.4%.

We divided the participants into categories of high or low BMI (≥25.0 kg/m^2^ or not), WC (≥85.0 cm or not), and VFA (≥100.0 cm^2^ or not), according to the cut off values for obesity and abdominal obesity used to assess metabolic syndrome in Japan, respectively [17, 18]. Furthermore, participants were divided into categories of high or low SFA (≥ 100.0 cm^2^ or not), to determine the impact of interaction effects between VFA and SFA on LECT2 levels. High BMI, WC, VFA, SFA was observed among 36 (25.2%), 64 (44.8%), 84 (58.7%), and 75 (52.4%) participants, respectively.

### CRF

CRF was assessed via a maximal graded exercise test using a cycle ergometer (Ergomedic 828E, Monark, Varberg, Sweden) and was quantified as peak oxygen uptake ($\dot{V}O_{2}\mathrm{peak}$). The graded cycle exercise began at a workload of 30–90 W, which was increased until the participant could no longer maintain a pedaling frequency of 60 rpm. The workload was increased by 15 W/min until the participants could no longer maintain the required pedalling speed of 60 rpm. The heart rate and the ratings of perceived exertion were monitored every minute during the exercise. During the incremental portion of the exercise test, the participant’s expired gas was collected and the O_2_ and CO_2_ concentrations were measured and averaged over 30-s intervals using an automated gas analyzing system (Aeromonitor AE-300, Minato Medical Science, Tokyo, Japan). The highest value of $\dot{V}O_{2}$ recorded during the exercise test was considered the $\dot{V}O_{2}\mathrm{peak}$.

### Blood pressure

Brachial SBP and DBP were measured using the oscillometric method (VaSera VS-1500N, Fukuda Denshi, Tokyo, Japan) among the participants at rest in the supine position [20].

### Collection and analysis of blood samples

The participants were instructed not to engage in any intensive exercise on the day before the blood sampling. Blood samples were collected between 8:30 and 10:00 AM after a 12-h overnight fast and then centrifuged at 3,000 rpm at 4°C for 15 min. The serum and plasma were collected and stored at –80°C until analysis. The serum enzymatic activities of aspartate aminotransferase (AST), alanine aminotransferase (ALT), and γ-glutamyl transferase (γ-GTP) and the concentrations of low-density lipoprotein (LDL) cholesterol, high-density lipoprotein (HDL) cholesterol, triglycerides, fasting glucose, and fasting insulin were determined at BML, Inc. (Tokyo, Japan). HOMA-IR was calculated from the fasting concentrations of plasma glucose and serum insulin as follows:

HOMA−IR=fastingglucose(mg/dL)×fastinginsulin(µU/mL)∕405

The plasma LECT2 concentration and the serum concentrations of tumor necrosis factor-alpha (TNF-α) and monocyte chemotactic protein-1 (MCP-1) were determined using a commercially available ELISA kit (LECT2: Ab-Match ASSEMBLY Human LECT2, Medical & Biological Laboratories, Nagoya, Japan; TNF-α: HSTA00D, R&D Systems, Inc., Minneapolis, MN, US; MCP1: DCP00, R&D Systems, Inc., Minneapolis, MN, US) according to the manufacturer’s instructions. The intra-assay coefficient of variation reported by the manufacturer was 2.6–3.2%, 3.1–8.7%, and 4.7–7.8% for LECT2, TNF-α, and MCP-1, respectively.

### Statistical analysis

Statistical analyses were performed using SPSS version 22.0 (SPSS, Inc., Chicago, IL, US) or R version 3.3.1 (R Development Core Team, 2016). The Kolmogorov-Smirnov test was performed to assess the normality of the data distribution, and several variables were log-transformed prior to analysis to obtain a normal distribution of values.

Comparisons of the differences in plasma LECT2 levels between two groups were analyzed using a Mann–Whitney U test. Correlations between the variables were determined by calculating Pearson’s correlation coefficients. Partial correlation analysis adjusted for age and BMI was also performed. We performed stepwise multiple linear regression analyses to identify the independent predictor of plasma LECT2 level. We selected age, alcohol intake, current or former smoking status, medication use (anti-hypertensive, lipid-lowering, gout suppressing, and prostatic hyperplasia drugs), and the variables that showed significant partial correlation with LECT2 after adjustment for age as the independent variables and LECT2 as the dependent variable. Because it was suggested that at least 10 participants per independent variables was required to minimize the bias [21], we selected the seven variables that showed the strongest correlation with LECT2 levels in addition to the dependent variables described above; therefore, a total of 14 variables were entered into the regression model. Multicollinearity among variables was examined by calculating the variance inflation factor, and we did not include the variables showing variance inflation factor values greater than 10 in the model.

To assess the effect of VFA on LECT2 levels in the participants stratified by BMI, WC, and SFA, we used two-way analysis of covariance (ANCOVA) (high and low VFA × high and low BMI, WC, or SFA) adjusted for age. A post hoc test with Bonferroni correction was used to identify significant differences among mean values if a significant interaction was identified.

We performed multiple logistic regression analyses to determine whether LECT2 is the independent predictor of the presence of metabolic syndrome and dyslipidemia, as well as whether LECT2 should be considered as a mediator of the association of VFA with the presence of metabolic syndrome and dyslipidemia. Model 1 included either LECT2 or VFA as an independent variable, while in model 2, LECT2 and VFA were simultaneously entered into the model. Both models were adjusted for age, alcohol intake, current or former smoking status, and anti-hypertensive (for metabolic syndrome) or lipid-lowering (for metabolic syndrome and dyslipidemia) drug use.

Receiver-operating characteristic (ROC) curves were plotted and the area under the curve (AUC) was calculated using pROC package in R [22] to evaluate the predictive values of LECT2 and VFA for dyslipidemia. Optimal cutoff values were set at the point on the ROC curve closest to the upper left corner of the graph.

Statistical power analyses were performed using G* power 3.1 [23]. The statistical power for correlation analyses were above 80% for *r* ≥ 0.24, at the two-tailed α = 0.05 level, for the 143 participants included in the study. Statistical power for linear regression analyses were above 80% to detect a medium effect size (f^2^ = 0.15) at the two-tailed α = 0.05 level, when 14 independent variables were included in the linear regression model.

All the measurements and calculated values are presented as means ± standard deviation for the normally distributed variables or as medians and interquartile range for the non-normally distributed variables. The statistical significance level was set at *P* < 0.05.

## Results

**Fig 1:**
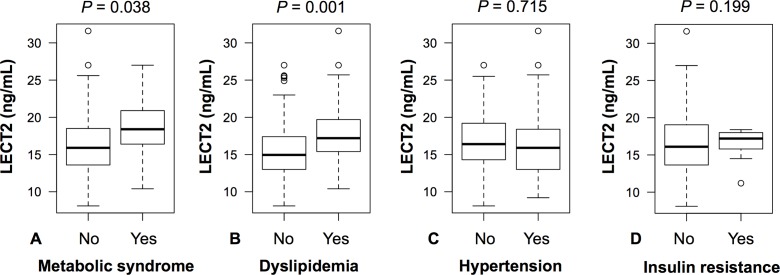
Differences in Plasma LECT2 levels Based on the Presence of (A) Metabolic Syndrome, (B) Dyslipidemia, (C) Hypertension, and (D) Insulin resistance. Box-plots show the median value and interquartile range of plasma LECT2 levels; open circles indicate outliers from 1.5- to 3.0-fold interquartile range.

The range of plasma LECT2 levels in the present study was 8.1–31.6 ng/mL. There were no extreme outliers with plasma LECT2 levels greater than 3.0-fold interquartile ranges below the first quartile or above the third quartile. The participants with metabolic syndrome had significantly higher levels of LECT2 than those without metabolic syndrome (Fig 1A). Participants with dyslipidemia also had higher levels of LECT2 than those without dyslipidemia (Fig 1B). Plasma LECT2 levels did not differ based on the presence of hypertension (Fig 1C) or insulin resistance (Fig 1D).

**Fig 2:**
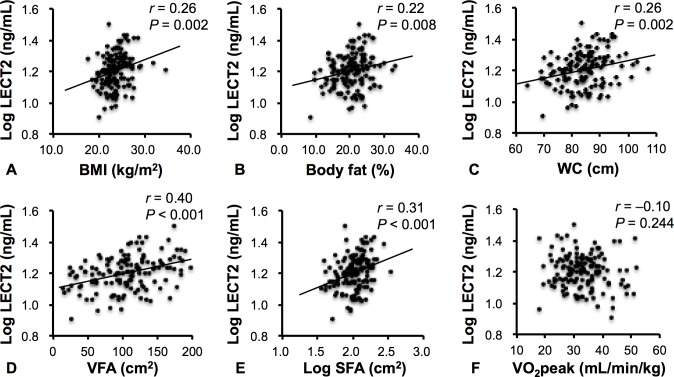
Correlations Between Plasma LECT2 Levels and BMI (A), Body Fat (B), WC (C), VFA (D), SFA (E), and $\dot{V}O_{2}\mathrm{peak}$ (F). LECT2 and SFA were log-transformed for analysis.

Fig 2 shows the correlation between plasma LECT2 levels and adiposity and fitness indices. Our cross-sectional study indicated that plasma LECT2 levels were positively correlated with BMI, body fat, WC, VFA, and SFA, but not significantly correlated with $\dot{V}O_{2}\mathrm{peak}$. Plasma LECT2 levels were also significantly correlated with glucolipid levels and liver enzyme levels such as ALT (*r* = 0.20, *P* = 0.015), HDL cholesterol level (*r* = –0.29, *P* = 0.001), LDL cholesterol level (*r* = 0.27, *P* = 0.001), and triglyceride level (*r* = 0.37, *P* < 0.001). Although LECT2 levels were not different between the presence and absence of insulin resistance, fasting insulin level (*r* = 0.24, *P* = 0.004) and HOMA-IR (*r* = 0.22, *P* = 0.010) were significantly correlated with LECT2 levels. Plasma LECT2 levels were not correlated with SBP (*r* = –0.11, *P* = 0.199), DBP (*r* = 0.00, *P* = 0.981), TNF-α (*r* = 0.11, *P* = 0.178), and MCP-1 (*r* = –0.08, *P* = 0.350).

Table 2 shows the results of the partial correlation analysis. The variables that were associated with LECT2 levels in the simple correlation analysis were also significantly correlated with LECT2 levels after adjustment for age. Although plasma LECT2 levels were not significantly correlated with $\dot{V}O_{2}\mathrm{peak}$, the levels were negatively correlated with $\dot{V}O_{2}\mathrm{peak}$ after adjustment for age. Because the stronger correlation between plasma LECT2 levels and VFA compared to other variables was observed, we examined the correlations between plasma LECT2 levels and other variables with further adjustment for VFA. The correlations between plasma LECT2 levels and all adiposity indices were not significant after adjustment for VFA (Table 2). Among the glucolipid levels and lipid enzymes, only serum levels of LDL cholesterol and triglycerides were significantly correlated with plasma LECT2 levels after adjustment for VFA. Additionally, the correlation between plasma LECT2 levels and VFA was examined when the participants were stratified by age, presence of dyslipidemia or hypertension, and by medication use. Plasma LECT2 levels were significantly correlated with VFA after adjustment for age regardless of: age (<65 years *r* = 0.45, *P* < 0.001; ≥65 years: *r* = 0.41, *P* = 0.001); the presence of dyslipidemia (*r* = 0.34, *P* = 0.009) versus non-dyslipidemia (*r* = 0.41, *P* < 0.001) or hypertension (*r* = 0.50, *P* < 0.001) versus normotension (*r* = 0.42, *P* = 0.001); and any medication use (*r* = 0.50, *P* < 0.001) versus no medication use (*r* = 0.39, *P* < 0.001).

**Table 2:** Correlations Between Plasma LECT2 Levels and Study Variables.

	LECT2 (age-adjusted)		LECT2 (age- and VFA-adjusted)	
	*R*	*P*	*R*	*P*
BMI	0.25	**0.003**	–0.04	0.656
Body fat	0.26	**0.002**	–0.03	0.729
WC	0.29	**0.001**	–0.04	0.612
VFA	0.43	**<0.001**		
SFA	0.30	**<0.001**	0.02	0.782
$\dot{V}O_{2}\mathrm{peak}$	–0.22	**0.008**	–0.09	0.289
SBP	–0.07	0.432	–0.14	0.093
DBP	0.04	0.658	–0.10	0.228
AST	–0.03	0.758	–0.06	0.471
ALT	0.17	**0.046**	0.05	0.580
γ-GTP	0.08	0.358	–0.11	0.184
HDL cholesterol	–0.26	**0.002**	–0.14	0.112
LDL cholesterol	0.28	**0.001**	0.26	**0.002**
Triglycerides	0.37	**<0.001**	0.26	**0.002**
Fasting glucose	–0.01	0.916	–0.05	0.547
Fasting insulin	0.24	**0.005**	0.09	0.283
HOMA-IR	0.23	**0.007**	0.08	0.328
TNF-α	0.12	0.137	0.08	0.379
MCP-1	–0.06	0.461	–0.11	0.190

SFA, SBP, DBP, AST, ALT, γ-GTP, HDL cholesterol, triglycerides, fasting insulin, HOMA-IR, TNF-α, MCP-1, and LECT2 were log-transformed for analysis. Bold numbers indicate statistical significance (*P* < 0.05).

To determine the independent predictor of plasma LECT2 levels, we performed a stepwise multiple linear regression analysis (Table 3). We included age, body fat, WC, VFA, SFA, HDL cholesterol, LDL cholesterol, triglycerides, alcohol intake, current or former smoking status, and medicine use (anti-hypertensive, lipid-lowering, gout suppressing, and prostatic hyperplasia drug use) as independent variables. In the best-fit model, VFA was most strongly associated with plasma LECT2 levels, whereas other adiposity indices were not independently associated with LECT2. Age, LDL cholesterol, and triglycerides levels were also significant predictors of plasma LECT2 levels. $\dot{V}O_{2}\mathrm{peak}$ was not independently associated with plasma LECT2 levels, and was excluded from the model.

**Table 3:** Stepwise Multiple Linear Regression Analysis to Determine the Independent Predictor of Plasma LECT2 Levels.

Dependent variables	Independent variables	*β*	*t*	*P*
LECT2	VFA	0.347	4.431	**<0.001**
	Age	-0.204	-2.756	**0.007**
	LDL cholesterol	0.177	2.287	**0.024**
	Triglycerides	0.180	2.194	**0.030**

*β*: Standardized coefficient. The model included body fat, WC, SFA, HDL cholesterol, HOMA-IR, alcohol intake, current or former smoking status, and drug use (lipid-lowering, anti-hypertensive, gout suppressing, and prostatic hyperplasia drug use) as independent variables. SFA, HDL cholesterol, triglycerides, and LECT2 were log-transformed for analysis. Bold numbers indicate statistical significance (*P* < 0.05). Model *r*^2^ = 0.280, *P* < 0.001.

**Fig 3:**
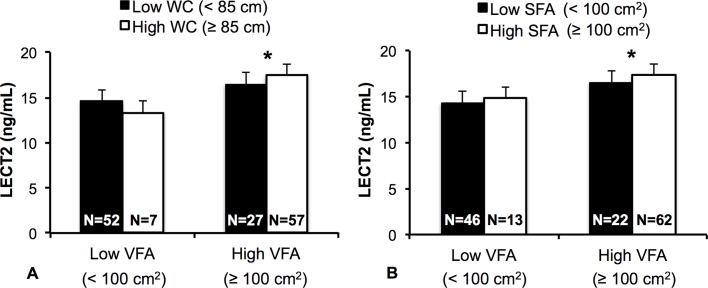
Differences in Plasma LECT2 Levels Between the Participants with High and Low VFA Stratified by High and Low WC (A) or SFA (B) Categories. Data are expressed as geometric mean and geometric standard deviation. Values in the bar graph represent the number of participants in each category. *P < 0.001 vs. low VFA.

We also assessed the effect of VFA on LECT2 levels in the participants stratified by BMI, WC, or SFA. Because only 3 participants with low VFA were classified into the high BMI category, stratified analysis was restricted to WC and SFA. Two-way ANCOVA, adjusted for age, detected no interaction effect between VFA and WC or VFA and SFA categories on plasma LECT2 levels (*P* = 0.316 versus *P* = 0.850, respectively). The main effect of VFA was significant (*P* < 0.001) and the participants with high VFA showed significantly higher LECT2 levels than the participants with low VFA, both in high and low WC or SFA categories (Fig 3A and 3B). Furthermore, the main effects of WC and SFA were not significant (*P* = 0.909 and *P* = 0.583, respectively) and plasma LECT2 levels were not different between the participants with high and low WC or SFA both in the high and low VFA categories.

Finally, we examined the relationship of LECT2 levels and VFA, with the presence of metabolic syndrome and dyslipidemia. In model 1, both plasma LECT2 levels and VFA were significantly associated with the presence of dyslipidemia and metabolic syndrome, respectively (Table 4). In model 2, plasma LECT2 levels were associated with the presence of dyslipidemia even after adjustment for VFA, whereas the association between VFA and dyslipidemia was attenuated after adjustment for LECT2 levels. On the contrary, plasma LECT2 levels were not associated with the presence of metabolic syndrome after adjustment for VFA, while the association between VFA and metabolic syndrome remained significant after adjustment for LECT2. To demonstrate the clinical significance of measuring circulating LECT2, we performed ROC analysis to examine whether plasma LECT2 levels can be used to discriminate the participants with and without dyslipidemia. The value of AUC for LECT2 levels was significant (AUC = 0.663; 95% CI: 0.572, 0.754; *P* = 0.001), and the optimal cutoff value was 16.5 ng/mL (sensitivity = 0.603, specificity = 0.667). Furthermore, we examined the practical value of LECT2 in addition to VFA for diagnosis of dyslipidemia. When LECT2 was added to the model, the AUC for VFA was improved from 0.656 (95% CI: 0.560, 0.740; *P* = 0.002) to 0.697 (95% CI: 0.610, 0.783; *P* < 0.001); however, the difference was not statistically significant (*P* = 0.097).

**Table 4:** Odds Ratio for Dyslipidemia and Metabolic Syndrome According to the Levels of LECT2 and VFA.

		Model 1		Model 2	
Dependent variables	Independent variables	OR (95% CI)	*P*	OR (95% CI)	*P*
Dyslipidemia	LECT2 (per 1 ng/mL increase)	1.18 (1.07–1.30)	**0.001**	1.14 (1.03–1.27)	**0.014**
	VFA (per 10 cm^2^ increase)	1.14 (1.04–1.25)	**0.004**	1.09 (0.98–1.20)	0.103
Metabolic syndrome	LECT2 (per 1 ng/mL increase)	1.19 (1.03–1.38)	**0.018**	1.10 (0.93–1.30)	0.265
	VFA (per 10 cm^2^ increase)	1.44 (1.17–1.79)	**0.001**	1.41 (1.13–1.75)	**0.002**

OR: odds ratio; CI: confidence interval. Model 1 included only one of either LECT2 or VFA as independent variable. Model 2 included both LECT2 and VFA as independent variables. All models adjusted for age, alcohol intake, current or former smoking status, anti-hypertensive drug use (for metabolic syndrome) and lipid-lowering drug use (for dyslipidemia and metabolic syndrome). Bold numbers indicate statistical significance (*P* < 0.05).

## Discussion

The present study demonstrated that VFA was the strongest predictor of plasma LECT2 levels, whereas other adiposity indices and CRF was not independently associated with plasma LECT2 levels in Japanese men. Furthermore, we also indicated that plasma LECT2 was a potential biomarker linking visceral fat accumulation to dyslipidemia.

Previous studies have already demonstrated that adiposity indices such as BMI and WC are positively correlated with circulating LECT2 levels [9, 24]; however, the relationships between body fat distributions such as the abdominal visceral and subcutaneous fat distributions and LECT2 levels have not been examined. The present study demonstrated that adiposity indices, except for VFA, were not associated with plasma LECT2 levels after adjustment for VFA. In addition, the participants with VFA less than 100 cm^2^ showed low LECT2 levels even if they had WC levels higher than the cut off values for abdominal obesity to assess metabolic syndrome in Japan or, SFA levels greater than 100 cm^2^. These results indicate that visceral fat accumulation significantly contributes to increased levels of LECT2. Although WC is a simple anthropometric measurement commonly used as an indicator of visceral obesity, it does not always accurately reflect VFA. In fact, 18.9% (n = 27) of individuals with VFA greater than 100 cm^2^ were not diagnosed as having visceral obesity when their abdominal fat was evaluated by measuring WC. Therefore, it is important to examine the relationship between the actual values of VFA and LECT2 levels to identify the primary contributor to increased LECT2 levels. On the contrary, the correlation between SFA and the LECT2 levels was no longer significant after adjustment for VFA. The positive correlation between SFA and VFA in the present study (*r* = 0.63, *P* < 0.001), reflects the effect of VFA on LECT2. These findings shed light as to why VFA, but not SFA, plays a crucial role in the development of various metabolic diseases.

The molecular mechanisms by which accumulation of visceral fat, but not subcutaneous fat, enhances liver *LECT2* expression remain unknown. Candidate factors linking visceral fat to LECT2 are adipokines, such as adiponectin, leptin, TNF-α, and MCP-1, which are derived from the visceral adipose tissue because these adipokines are mainly secreted from visceral adipose tissue rather than subcutaneous adipose tissue and were demonstrated to be key mediators linking visceral obesity to metabolic diseases [25]. In the present study, we examined the relationship of LECT2 with TNF-α and MCP-1, which are pro-inflammatory adipokines involved in the development of metabolic diseases [25]. However, neither adipokines were associated with the LECT2 levels, suggesting that pro-inflammatory adipokines are not mediators linking visceral fat to LECT2. Nonetheless, it has been demonstrated that adipokines that regulate energy homeostasis, such as adiponectin and leptin, stimulate phosphorylation and activation of liver AMPK [26, 27]; therefore, visceral obesity may inhibit liver AMPK activity via decreases in adiponectin and leptin signaling, resulting in the upregulation of *LECT2* in the liver. However, we could not measure adiponectin and leptin levels in our study owing to lack of blood samples; therefore, further investigation is needed to clarify the role of these adipokines in the regulation of LECT2.

Although many epidemiological studies have revealed that higher levels of CRF are associated with lower incidence of type 2 diabetes and better insulin sensitivity [11–14], the underling mechanisms have not been fully elucidated. Because Lan et al. reported that acute exercise in mice increased the phosphorylation and activity of liver AMPK, thereby decreasing *Lect2* expression in the liver [9], LECT2 was hypothesized to be a candidate hepatokine that mediates the relationship between regular exercise or high CRF and improved insulin resistance and type 2 diabetes prevention. However, we did not detect an independent association between CRF and plasma LECT2 levels in humans. Although $\dot{V}O_{2}\mathrm{peak}$ was negatively correlated with plasma LECT2 levels after adjustment for age and BMI (*r* = –0.18, *P* = 0.035), the association was no longer significant when we further adjusted for VFA (*r* = –0.09, *P* = 0.293). A previous study demonstrated that CRF is negatively associated with VFA independently of BMI [28]; therefore, the association between $\dot{V}O_{2}\mathrm{peak}$ and LECT2 levels might be mediated by VFA in the present study. Although liver AMPK was activated by chronic exercise in rodents [29, 30], the effects of regular exercise or high CRF on liver AMPK have not been examined in humans. Another study demonstrated that the phosphorylation and activity of skeletal muscle AMPK in the resting state were not different between exercise-trained with high CRF men and untrained men [31]. Therefore, we can speculate that CRF in humans does not affect AMPK activity in the liver as well as in the skeletal muscle, thereby not affecting LECT2 levels.

Although LECT2 has been suggested to play a role in obesity-induced insulin resistance, our cross-sectional study in Japanese men demonstrated that HOMA-IR was not independently associated with plasma LECT2 levels. The cross-sectional study performed by Okumura et al. demonstrated that HOMA-IR was significantly associated with LECT2 levels in Japanese women but not in men [24]. Therefore, LECT2 may influence insulin resistance specifically in women. On the other hand, Okumura et al. reported that the level of serum triglycerides was an independent predictor of LECT2 in men [24]. The present study also showed that blood lipids such as triglycerides and LDL cholesterol levels were independently associated with LECT2, suggesting that LECT2 plays a role in the development of dyslipidemia rather than insulin resistance in male participants. The results of the logistic regression analyses also demonstrated that LECT2 was the independent predictor of dyslipidemia. Furthermore, the lack of association between VFA and dyslipidemia after adjustment for LECT2 suggests that the relationship between VFA and dyslipidemia is partially mediated by LECT2. Because it has been confirmed that activation of liver AMPK enhances fatty acid oxidation and inhibits triglycerides and cholesterol synthesis [32], LECT2 may play a role in visceral fat-induced inactivation of liver AMPK, and therefore be associated with dyslipidemia. Furthermore, the ROC analysis demonstrated that plasma LECT2 could be used as a biomarker to diagnose dyslipidemia in men, even though including LECT2 in addition to VFA in the regression model did not significantly increase the predictive value. The optimal cutoff value for plasma LECT2 levels for the diagnosis of dyslipidemia was 16.5 ng/mL, suggesting that variation in plasma LECT2 levels, regardless of extremity, have clinical significance.

Aging is the primary predictor of various metabolic diseases. However, our results from multiple regression analysis showed that age was a negative predictor of plasma LECT2 levels. Okumura et al. also reported that age was negatively associated with LECT2 levels in Japanese men [24]. Therefore, although aging enhances visceral fat accumulation that results in LECT2 induction, aging per se does not likely increase LECT2 levels in humans.

The present study has several limitations. First, the sample size of our study was relatively small, which might have led to a type 2 error. Second, our study included only male participants. Previous studies indicated that the strength of associations between metabolic parameters and circulating LECT2 levels are different between men and women. The relationship of adiposity indices and CRF on LECT2 levels should therefore be confirmed in female participants. Third, we did not evaluate hepatic fat content despite a previous study that showed fatty liver to be associated with high levels of circulating LECT2 [24]. The associations of adiposity indices and CRF with plasma LECT2 should be re-examined after taking into account hepatic fat content as a covariate. Finally, although the present cross-sectional study demonstrated LECT2 was a significant predictor of dyslipidemia, it is still unclear whether LECT2 is also associated with development of dyslipidemia and the incidence of coronary heart diseases. Longitudinal studies are needed to establish the clinical significance of LECT2 measurements for the prediction of dyslipidemia and its related diseases.

In conclusion, the present study indicates that VFA is the strongest predictor of plasma LECT2 levels in Japanese men. Our data also indicate that LECT2 is a potential biomarker linking visceral obesity to dyslipidemia.

## References

[1] Iroz A, Couty JP, Postic C. Hepatokines: unlocking the multi-organ network in metabolic diseases. Diabetologia. 2015;58(8):1699–703. 10.1007/s00125-015-3634-4 26032022

[2] Nishimura T, Nakatake Y, Konishi M, Itoh N. Identification of a novel FGF, FGF-21, preferentially expressed in the liver. Biochim Biophys Acta. 2000;1492(1):203–6. 10858549 10.1016/s0167-4781(00)00067-1

[3] Denecke B, Graber S, Schafer C, Heiss A, Woltje M, Jahnen-Dechent W. Tissue distribution and activity testing suggest a similar but not identical function of fetuin-B and fetuin-A. Biochem J. 2003;376(Pt 1):135–45. PubMed Central PMCID: PMCPMC1223762. 10.1042/BJ20030676 12943536 PMC1223762

[4] Burk RF, Hill KE. Selenoprotein P: an extracellular protein with unique physical characteristics and a role in selenium homeostasis. Annu Rev Nutr. 2005;25:215–35. 10.1146/annurev.nutr.24.012003.132120 16011466

[5] Fisher FM, Kleiner S, Douris N, Fox EC, Mepani RJ, Verdeguer F, et al FGF21 regulates PGC-1alpha and browning of white adipose tissues in adaptive thermogenesis. Genes Dev. 2012;26(3):271–81. PubMed Central PMCID: PMCPMC3278894. 10.1101/gad.177857.111 22302939 PMC3278894

[6] Misu H, Takamura T, Takayama H, Hayashi H, Matsuzawa-Nagata N, Kurita S, et al A liver-derived secretory protein, selenoprotein P, causes insulin resistance. Cell Metab. 2010;12(5):483–95. 10.1016/j.cmet.2010.09.015 21035759

[7] Pal D, Dasgupta S, Kundu R, Maitra S, Das G, Mukhopadhyay S, et al Fetuin-A acts as an endogenous ligand of TLR4 to promote lipid-induced insulin resistance. Nat Med. 2012;18(8):1279–85. 10.1038/nm.2851 22842477

[8] Potthoff MJ, Inagaki T, Satapati S, Ding X, He T, Goetz R, et al FGF21 induces PGC-1alpha and regulates carbohydrate and fatty acid metabolism during the adaptive starvation response. Proc Natl Acad Sci U S A. 2009;106(26):10853–8. PubMed Central PMCID: PMCPMC2705613. 10.1073/pnas.0904187106 19541642 PMC2705613

[9] Lan F, Misu H, Chikamoto K, Takayama H, Kikuchi A, Mohri K, et al LECT2 functions as a hepatokine that links obesity to skeletal muscle insulin resistance. Diabetes. 2014;63(5):1649–64. 10.2337/db13-0728 24478397

[10] Fox CS, Massaro JM, Hoffmann U, Pou KM, Maurovich-Horvat P, Liu CY, et al Abdominal visceral and subcutaneous adipose tissue compartments: association with metabolic risk factors in the Framingham Heart Study. Circulation. 2007;116(1):39–48. 10.1161/CIRCULATIONAHA.106.675355 17576866

[11] Clausen JO, Borch-Johnsen K, Ibsen H, Bergman RN, Hougaard P, Winther K, et al Insulin sensitivity index, acute insulin response, and glucose effectiveness in a population-based sample of 380 young healthy Caucasians. Analysis of the impact of gender, body fat, physical fitness, and life-style factors. J Clin Invest. 1996;98(5):1195–209. PubMed Central PMCID: PMCPMC507542. 10.1172/JCI118903 8787683 PMC507542

[12] Kasa-Vubu JZ, Lee CC, Rosenthal A, Singer K, Halter JB. Cardiovascular fitness and exercise as determinants of insulin resistance in postpubertal adolescent females. J Clin Endocrinol Metab. 2005;90(2):849–54. 10.1210/jc.2004-0455 15572432

[13] Sawada SS, Lee IM, Muto T, Matuszaki K, Blair SN. Cardiorespiratory fitness and the incidence of type 2 diabetes: prospective study of Japanese men. Diabetes Care. 2003;26(10):2918–22. 14514602 10.2337/diacare.26.10.2918

[14] Sui X, Hooker SP, Lee IM, Church TS, Colabianchi N, Lee CD, et al A prospective study of cardiorespiratory fitness and risk of type 2 diabetes in women. Diabetes Care. 2008;31(3):550–5. PubMed Central PMCID: PMCPMC3410433. 10.2337/dc07-1870 18070999 PMC3410433

[15] Tanisawa K, Ito T, Sun X, Cao ZB, Sakamoto S, Tanaka M, et al Polygenic risk for hypertriglyceridemia is attenuated in Japanese men with high fitness levels. Physiol Genomics. 2014;46(6):207–15. 10.1152/physiolgenomics.00182.2013 24474445

[16] Tanisawa K, Ito T, Sun X, Ise R, Oshima S, Cao ZB, et al Strong influence of dietary intake and physical activity on body fatness in elderly Japanese men: age-associated loss of polygenic resistance against obesity. Genes Nutr. 2014;9(5):416 PubMed Central PMCID: PMCPMC4172647. 10.1007/s12263-014-0416-4 25030601 PMC4172647

[17] Matsuzawa Y. Metabolic syndrome—definition and diagnostic criteria in Japan. J Atheroscler Thromb. 2005;12(6):301 16394611 10.5551/jat.12.301

[18] Examination Committee of Criteria for 'Obesity Disease' in J, Japan Society for the Study of O. New criteria for 'obesity disease' in Japan. Circ J. 2002;66(11):987–92. 12419927 10.1253/circj.66.987

[19] Taniguchi H, Tanisawa K, Sun X, Cao ZB, Oshima S, Ise R, et al Cardiorespiratory fitness and visceral fat are key determinants of serum fibroblast growth factor 21 concentration in Japanese men. J Clin Endocrinol Metab. 2014;99(10):E1877–84. 10.1210/jc.2014-1877 25013999

[20] Tanisawa K, Ito T, Sun X, Kawakami R, Oshima S, Gando Y, et al Cardiorespiratory Fitness is a Strong Predictor of the Cardio-ankle Vascular Index in Hypertensive Middle-aged and Elderly Japanese Men. J Atheroscler Thromb. 2015;22(4):379–89. 10.5551/jat.25098 25342380

[21] Harrell FE. Regression Modeling Strategies: Springer-Verlag: New York; 2001.

[22] Robin X, Turck N, Hainard A, Tiberti N, Lisacek F, Sanchez JC, et al pROC: an open-source package for R and S+ to analyze and compare ROC curves. BMC Bioinformatics. 2011;12:77 PubMed Central PMCID: PMCPMC3068975. 10.1186/1471-2105-12-77 21414208 PMC3068975

[23] Faul F, Erdfelder E, Buchner A, Lang AG. Statistical power analyses using G*Power 3.1: Tests for correlation and regression analyses. Behav Res Methods. 2009;41(4):1149–60. 10.3758/BRM.41.4.1149 19897823

[24] Okumura A, Unoki-Kubota H, Matsushita Y, Shiga T, Moriyoshi Y, Yamagoe S, et al Increased serum leukocyte cell-derived chemotaxin 2 (LECT2) levels in obesity and fatty liver. Biosci Trends. 2013;7(6):276–83. 24390366

[25] Ouchi N, Parker JL, Lugus JJ, Walsh K. Adipokines in inflammation and metabolic disease. Nat Rev Immunol. 2011;11(2):85–97. PubMed Central PMCID: PMCPMC3518031. 10.1038/nri2921 21252989 PMC3518031

[26] Yamauchi T, Kamon J, Minokoshi Y, Ito Y, Waki H, Uchida S, et al Adiponectin stimulates glucose utilization and fatty-acid oxidation by activating AMP-activated protein kinase. Nat Med. 2002;8(11):1288–95. 10.1038/nm788 12368907

[27] Miyamoto L, Ebihara K, Kusakabe T, Aotani D, Yamamoto-Kataoka S, Sakai T, et al Leptin activates hepatic 5'-AMP-activated protein kinase through sympathetic nervous system and alpha1-adrenergic receptor: a potential mechanism for improvement of fatty liver in lipodystrophy by leptin. J Biol Chem. 2012;287(48):40441–7. PubMed Central PMCID: PMCPMC3504759. 10.1074/jbc.M112.384545 23024365 PMC3504759

[28] Wong SL, Katzmarzyk P, Nichaman MZ, Church TS, Blair SN, Ross R. Cardiorespiratory fitness is associated with lower abdominal fat independent of body mass index. Med Sci Sports Exerc. 2004;36(2):286–91. 10.1249/01.MSS.0000113665.40775.35 14767252

[29] Takekoshi K, Fukuhara M, Quin Z, Nissato S, Isobe K, Kawakami Y, et al Long-term exercise stimulates adenosine monophosphate-activated protein kinase activity and subunit expression in rat visceral adipose tissue and liver. Metabolism. 2006;55(8):1122–8. 10.1016/j.metabol.2006.04.007 16839850

[30] Yi X, Cao S, Chang B, Zhao D, Gao H, Wan Y, et al Effects of acute exercise and chronic exercise on the liver leptin-AMPK-ACC signaling pathway in rats with type 2 diabetes. J Diabetes Res. 2013;2013:946432 PubMed Central PMCID: PMCPMC3877642. 10.1155/2013/946432 24455748 PMC3877642

[31] Nielsen JN, Mustard KJ, Graham DA, Yu H, MacDonald CS, Pilegaard H, et al 5'-AMP-activated protein kinase activity and subunit expression in exercise-trained human skeletal muscle. J Appl Physiol (1985). 2003;94(2):631–41. 12391032 10.1152/japplphysiol.00642.2002

[32] Viollet B, Foretz M, Guigas B, Horman S, Dentin R, Bertrand L, et al Activation of AMP-activated protein kinase in the liver: a new strategy for the management of metabolic hepatic disorders. J Physiol. 2006;574(Pt 1):41–53. PubMed Central PMCID: PMCPMC1817784. 10.1113/jphysiol.2006.108506 16644802 PMC1817784

